# Japanese International Medical Graduates and entrance into US clinical training: Challenges and methods to overcome them

**DOI:** 10.1002/jgf2.332

**Published:** 2020-05-14

**Authors:** Brian S. Heist, Haruka Matsubara Torok

**Affiliations:** ^1^ University of Pittsburgh School of Medicine Pittsburgh Pennsylvania USA; ^2^ University of Minnesota Medical School Minneapolis Minnesota USA

**Keywords:** medical education, medical migration, postgraduate medical education, qualitative research

## Abstract

**Introduction:**

Entering US clinical training requires completing requirements and navigating an application process differing from the Japanese system. Additionally, increases to the number of US medical school graduates have increased competition for US residency positions. We examined profiles of Japanese International Medical Graduates (IMGs) who completed US clinical training, the timelines to securing US clinical positions, and the greatest challenges during this process and methods to overcome them.

**Methods:**

Individual semistructured interviews were conducted with 35 purposively sampled Japanese IMGs. We performed exploratory thematic analysis using iterative data collection and constant comparison.

**Results:**

Twenty percent of participants lived in a native English‐speaking country during childhood. The United States Medical Licensing Examinations were completed at ages 25‐40 years. Challenges were categorized as: (1) English communication, (2) understanding the application process, (3) motivation to persevere through the process, (4) time management to complete Educational Commission for Foreign Medical Graduates requirements, (5) receiving letters of recommendation and overcoming competition for US residency positions, (6) financial cost of the process. Pragmatic generally self‐dependent methods helped overcome challenges 1‐4 and 6. Participants detailed personal or, more commonly, institutional connections to US training programs required to overcome challenge 5.

**Conclusions:**

Japanese IMGs pursue US clinical training from diverse backgrounds commonly without the advantage of prior English fluency. Amidst increased competition internationally to enter US residency coupled with cultural and linguistic differences making this challenge often greater for Japanese IMGs, the competition to participate in institutionalized connections to US training programs is anticipated to increase.

## Graphical abstract

Individual interviews were conducted with 35 Japanese International Medical Graduates who had completed US clinical training, in order to understand the profiles of Japanese physicians who complete US training, the timelines to securing US clinical training positions, and their greatest challenges during this process and methods to overcome them. Main themes include that Japanese IMGs pursue US clinical training from diverse backgrounds commonly without the advantage of prior English fluency. Six categories of challenges were identified, including obtaining letters of recommendation which participants explained required personal or, more commonly, institutional connections to US training programs.

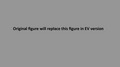

## 1 INTRODUCTION

Internationally, medical education is rapidly evolving in response to globalization and changing societal needs.^1^ American medical education is directly or indirectly influential to Japanese medical education, currently especially in emerging disciplines such as primary care medicine, geriatrics, palliative care, and infectious diseases.^2^, ^3^, ^4^ In turn, opportunity for some Japanese physicians to pursue US clinical training is important.

Entering US clinical training requires completing ECFMG (Educational Commission for Foreign Medical Graduates) certification and applying via the ERAS (Electronic Residency Application Service) process, which differ significantly from Japanese systems.^5^, ^6^ Distinctions include the submission of a portfolio including examination scores, faculty evaluations, and letters of recommendation in the American application process.^6^ Additionally, the ECFMG has mandated that applicants graduate from accredited medical schools as of 2023.^7^ Japan is meeting this requirement through establishment and ECFMG recognition of the JACME (Japan Accreditation Council for Medical Education) in 2017, followed by steady accreditation of medical schools across Japan.^8^, ^9^

Competition to enter US residency is impacted by the demand and supply for residency positions, and both are changing. Motivated by concerns of a future doctor shortage in the United States, from 2002‐2003 to 2018‐2019 the number of US first‐year allopathic medical school students and osteopathic medical students increased by 31% to 21 622 students and by 164% to 8124 students, respectively.^10^ Allopathic medical students receive the conventional Doctorate of Medicine (MD), while osteopathic medical students receive a Doctorate of Osteopathy (DO) in a long‐standing American physician training landscape that recognizes these slightly differing philosophies.^11^ Osteopathic medical students are eligible to take the USMLE and over half matriculate to allopathic residency training programs.^12^ During the above interim, the number of US residency positions has increased, but at a slower rate than the applicant population.^10^ These developments have increased competition for residency, with manifestations including a greater than 70% increase in the number of applications per residency program.^13^

The number of US residency positions exceeds the number of US medical school graduates, meaning there is ample US training opportunity for IMGs (International Medical Graduates).^14^ Nonetheless, the competition is stiff. IMGs perform significantly better on the USMLE (United States Medical Licensing Examination) but experience a 51% residency match rate, 35% lower than for US graduates.^14^, ^15^ These statistics are roughly unchanged since 2002,^14^, ^16^ but because of the increase in applications, residency directors are changing how they screen applicants. Changes include eliminating a holistic review of the applications and raising requirements for applicants invited to interviews.^13^ In some cases, residency programs that previously commonly accepted IMGs are now filled by US graduates.

Compared to most IMGs, Japanese face steeper barriers to entering and succeeding in US training, including linguistic distance and the absence of a culture of migration.^4^ In these global and local contexts, understanding the challenges and successes of Japanese physicians who have navigated US clinical training is increasingly relevant. Guidebooks exist for Japanese trainees interested in pursuing US training,^17^ but a data‐driven inductive study has not been performed. In this study of Japanese physicians who have completed US clinical training, we examine the greatest challenges to securing US clinical training positions and methods to overcome them. Relatedly, we examine educational and linguistic backgrounds to help develop profiles of this physician population.

## 2 MATERIALS AND METHODS

This report is complemented by a separate report from the same study that examines challenges for Japanese IMGs during US clinical training.^18^ Identical participants and methods were used, and the data were collected and analyzed simultaneously.

### 2.1 Study approach

A qualitative study design, specifically Constructivist exploratory thematic analysis,^19^ was selected given our interest in developing a conceptual understanding of which Japanese physicians become US clinically trained and how they achieve this goal. Constructivist methodology recognizes that data collection and interpretation are influenced by researchers' prior knowledge of the subject matter.^20^ The primary investigator completed all medical trainings in the United States but worked at a Japanese residency program for 3 years prior to commencing this study. The secondary investigator is a Japanese IMG. Both investigators have completed fellowships in medical education with coursework in qualitative methodology, and the primary investigator has previously conducted qualitative research. Both investigators are employed at US teaching institutions as clinician educators and have contributed to the entrance into and education during US residency for IMGs from many countries including Japan.

### 2.2 Participant sampling

Japanese IMGs working in the United States and Japan were purposively sampled with the goal of identifying diverse experiences. In the absence of a comprehensive database of Japanese IMGs, we identified potential participants by (a) asking participants to suggest other potential participants, optimally with experiences contrasting their own, and (b) requesting names of Japanese graduates from training programs in the United States and Japan who had educated Japanese IMGs. Inclusion criteria were graduation from a Japanese medical school and completion of US clinical training within one to fifteen years at time of interview. Additionally, a participant group of approximately 50% returnees to Japan and 50% practitioners in the United States was targeted. These parameters were chosen because separate work examines experiences after US training. Thirty‐five of 39 contacted physicians agreed to participate. Each participant provided informed consent. One or both investigators had prior relationships with eleven of the participants but had not previously discussed the issues in this study. The study was approved by the Institutional Review Board at the University of Pittsburgh Medical Center.

### 2.3 Data collection and analysis

We performed individual semistructured interviews in person, via phone, or via Skype from February 2013 until October 2015. Several days beforehand, participants received a question guide to allow reflection on the subject matter. Interviews were transcribed verbatim by a professional transcriptionist. Because the research was based at a US institution with local funding, hiring a transcriptionist and documenting expenses were simplified by performing the interviews in English. The investigators shared the interviewing responsibilities. The first six interviews were independently analyzed using open coding to create an initial codebook. Subsequent interviews and coding progressed in an iterative fashion such that findings from earlier interviews informed probing questions in later interviews. The primary investigator coded all transcripts. The secondary investigator independently coded seven transcripts and reviewed all of the primary investigator's coded transcripts and memos. We discussed our findings to resolve any disagreements and conducted interviews until themes were saturated. The software Atlas.ti 7.0 (Scientific Software) assisted the coding process.

In this study, participants were asked closed‐ended questions about their childhood English exposure, medical school experiences including international electives, timing of USMLE testing, and age upon entering US training. Participants were asked open‐ended questions about (a) the most challenging parts of the process to become a resident physician in the United States, (b) how they overcame those challenges, and (c) if there were people who helped along the way during their preparation. Follow‐up questions probed replies to the closed‐ended and open‐ended questions, and per the iterative relationship with data interpretation described above, were influenced by preceding interviews.

To enhance readability, we performed minor grammar editing of quotations. We confirmed the themes with two participants as a validity check.

## 3 RESULTS

### 3.1 Participant characteristics

Of the 35 participants, 80% (28/35) never lived in a native English‐speaking country during childhood. The participants attended 23 distinct medical schools. Roughly half of them participated in a clinical elective in the United States and/or observership at a US Naval Hospital in Japan (USNH). The participants completed the USMLE examinations most commonly during postgraduate year (PGY) 1‐2 and entered US training most commonly at age 28‐30 years, but the ranges were from the 5th year of medical school to PGY 15 and age 25‐40 years, respectively. Table 1 contains further details. With two exceptions, participants completed USMLE Step examinations within 2 years of taking Step 1. No difference in the timing of the examination was observed between participants who completed medical school recently vs remotely. Each participant was assigned a number (1‐35), which accompanies each quotation; participants 1‐19 and 20‐35 were employed in Japan and the United States, respectively, at the time of the interview.

**Table 1:** Characteristics of study participants

Characteristic	Participants (n = 35)
Male gender	24
Childhood residency^a^ in a native English‐speaking country	
None	28
<1 y	1
1‐2 y	3
>2 y	3
Nonmedical studies between high school and med school	2
Type of Japanese medical school attended	
National (16 distinct schools)	27
Public (3 distinct schools)	4
Private (4 distinct schools)	4
Educational or clinical elective in a native English‐speaking country during medical school	15
1‐wk observation at United States Naval Hospital in Japan	5
Timing for completion of USMLE Step examinations	
Med school year 5‐6	7
Postgraduate year 1‐2	15
Postgraduate year 3‐4	4
Postgraduate year 5‐6	4
Postgraduate year 7 or later	5
Age upon commencing US training	
25‐27 y	7
28‐30 y	15
31‐33 y	7
34‐36 y	2
37‐39 y	3
>39 y^b^	1

Abbreviation: USMLE, United States Medical Licensing Examination.

^a^ Defined as duration over 2 mo during age 2‐18 y.

^b^ One participant, who was age 40 upon commencing US training, bypassed US residency and entered directly in a US clinical fellowship.

### 3.2 Challenges and methods to overcome them during preparation for US residency

Six themes emerged among the challenges encountered by participants in their preparation for US residency: (1) English communication; (2) understanding the application process; (3) motivation to persevere through the process; (4) time management to complete the ECFMG requirements; (5) after completing the ECFMG requirements, receiving letters of recommendation and overcoming the intense competition for US residency positions; and (6) financial cost of the entire process. Participants described methods used to overcome each challenge. Table 2 contains representative quotes for the first four challenges.

**Table 2:** Challenges and methods to overcome them during preparation for US residency

Challenge	Methods to overcome challenge	Representative quotes
English	Extra instruction	I bought a 6 month English conversation school package… I went so frequently that I finished [the package] within a month. (6)
	Self‐learning	[While I was a full‐time resident physician in Japan,] when I got home I listened to or watched English educational programs, half an hour or one hour every day. (2)
	Clinical experiences in the United States	My externship at [a US teaching hospital] definitely helped my speaking skills. (4)
Understanding the application process	Senior connections	[My Japanese residency program] has a strong alumni group [who] always tried to help, like, “Oh, you're interested in this so you should talk to this person.” I had many connections. (34)
		[As I prepared for ECFMG certification] I called those three graduates [of my medical school who pursued US training] and asked more specific questions about… obtaining a residency position in the US (4)
		[My mentor, a US trained Japanese IMG,] tried to teach me English and medical knowledge, especially, US‐style medical knowledge. For one year he gave me many opportunities to present patients in front of US physicians… Without him, I definitely couldn't get into [US residency.] (14)
Motivation	Being among others with same goal	I had many residents [at my Japanese residency program] who thought about the same things, so we just encouraged each other. If I were doing residency at a university hospital in Japan, there would be nobody to share my thought with, so it would be very tough. (22)
Time management	Study plan during residency	I slept less, and [took advantage of lighter rotations].… I studied from evening until morning. And then in the morning, I would go to the [clinical ward] and work with the attending. By noon almost all of my work was done, and I would sleep until evening and start studying again. That was my cycle. (3)
		Balancing studying and work was difficult. I had patients that needed attention and I also needed time to study for USMLE. That made me, basically, sleep less… 4 hours per day for one and a half months. (15)
	Lightening work schedule	I realized it was almost impossible to prepare for the USMLE while working in [my current busy] hospital. I had either quit and work part‐time [somewhere else] while preparing for the USMLE or give up on United States residency. So I worked part‐time… maybe 3 times a week, and the rest of the time, I just concentrated on studying for the USMLE, going to private English lessons and conversation school… for maybe 9 months or so. (16)
	Test prep courses	Fortunately, Kaplan was a walkable distance from my med school…. I made so many friends [with classmates] there. (34)
		During my vacation [in residency] I went to New Jersey, took that one‐week intensive Kaplan course, and [then] moved to Philadelphia and took my Step Two CS. (21)

Regarding the fifth challenge, participants highlighted the challenge of being an IMG competing with domestic applicants.

[Selection committees] looked [at my application and probably thought] “Okay, she's an IMG, and her [USMLE] scores aren't that great, and then automatically put my file into another box.” (32)

Participants explained the need for letters of recommendation from US faculty and the consequent need for connections.

I went to multiple [US teaching hospitals for] externships. In order to match [into a US residency program], I needed a letter of recommendation from somebody inside. (10)

Most participants described personal and commonly institutional connections that enabled their entrance into US residency.

You need recommendation [letters] and you need some kind of connection with an American hospital, like the University of Pittsburgh and Teine [Keijinkai Hospital], Noguchi Foundation, or Tokyo Marine Insurance Company. Those are the realistic options for Japanese doctors. Getting into [US] residency without those connections would be very tough. [For example,] my friend who eventually got into the residency in Hawaii [through a connection], first tried [independently] for two consecutive years‐ he applied to 450 programs and got nothing. I think that's the reality. (13)

Participants most commonly reported using the N program (sponsored by the Tokyo Marine Insurance Company) or the Noguchi Institute, and sometimes both. They explained that the N program offers several prematch positions, primarily in internal medicine, at its affiliate, the Beth Israel Hospital in New York City. The Noguchi Institute offers observerships at two US training hospitals, including the University of Hawaii, through which numerous participants described receiving invitations to interview for residency positions. Some of these interviews resulted in prematch residency position offers.

In addition to the institutional bridges to US residency listed by Participant 13, numerous participants described the value of the 1‐year internship at a USNH in Japan; participants received opportunity to improve their fluency in English and American medical practice while also studying for USMLE examinations, letters of recommendation from American physicians, and sometimes opportunity for externships at US teaching hospitals.

If you go outside, it's Japan, but within the hospital, that's all the US, so it's very easy for us Japanese to get used to the US medical system and improve your communication. It's a perfect setting. (12)

Finally, several participants explained making their own connections, either while in Japan, or more often in the case of non–primary care‐related fields, moving to the United States and then making connections while pursing research to bolster their resumes. Table 3 lists the connections used to enter US residency with representative quotes.

**Table 3:** Connections used to enter US Residency

Connection	Representative quotes
N program—Beth Israel Hospital in New York City	I [entered US residency] through the N Program. I was verbally given a position before the match. I spoke English well enough, and they thought my personality would fit. (9)
	My [USMLE] scores were low, so I would not have made it here unless I was picked up by the N program. But they looked at my research track record and they liked it. (26)
Noguchi Medical Institute—Univ. of Hawaii	Five to ten [Noguchi Medical Institute members] can go to the US as externs [annually], and that is how [the Institute] helps us experience medicine in the US without an American medical license. (18)
	I had an observership [at U. Hawaii] through the Noguchi Medical Institute. I was applying to US Residency at the time, and then two weeks after coming back to Japan, they offered me an extra‐match position. (6)
USNH—Univ. of Hawaii	[During my internship] at the USNH, the Program Director somehow knew the Director of Medical Education of Internal Medicine Program at the University of Hawai'i… [They] set up an [affiliation] so interns at the Naval Hospital can spend a month at University of Hawai'i…. So, I went there… and received an interview… and I pre‐matched there. (11)
	During my PGY‐4 year, I went to the [US] Naval Hospital in Okinawa and there I could make time to study for [the USMLE] and also for an externship [at a hospital in the US] and some interviews, so it was good. Without that, [entering US residency] would have been more difficult. (17)
Teine Keijinkai Hospital—Univ. of Pittsburgh	[I did my Japanese residency at TKH] because they send residents to Pittsburgh, that's why. (22)
	[The internal medicine residency program director] from Pittsburgh came to TKH to see how we were doing. He observed me doing a history and physical, we spoke for a while, and I think he liked me, so as he was leaving he was like “Okay, pass the USMLE and I'll accept you [into residency in Pittsburgh].” (21)
Making one's own connections in Japan	The vice president of the University of Hawaii visited my [Japanese residency program]. After his lecture I went to his room and asked him if I can do an observership. He said OK. So I went to Hawaii for two weeks, and during that time, I tried to identify who was on the resident selection committee. I found someone who was not a program director, but who had power. So I wrote a very good progress note on his patient. And he was like, “Oh, who is this intern? His note is really good.” And I replied, “Oh, that's me.” And then I asked him for an externship opportunity. The next year I [returned] for the externship and he liked me, and I received an out‐of‐match position. (19)
Improving CV and establishing connections in the United States	I went to [a prestigious US medical institution to pursue] research, hoping that may increase my chance of entering US residency. After three years, I felt I had [a CV adequate] to get into an American surgical training program. [Even then] I found that obtaining a categorical [residency position] was almost impossible. I had good letters from an assistant professor there, etc and I applied for preliminary [residency positions] and matched into a general surgery preliminary year program [elsewhere]. However, I was not able to start [then] because my J1 visa was for research [purposes only.] (29)
	I submitted [my application] to all the programs in the United States, and received 3 interview invitations, including [where I had been working already.] When I mentioned to [the department chair] that I was heading to [another city] for an interview, he kind of looked at me and then suggested, “Well, you don't want to do that.” I asked “Why?” and then he replied, “Let me talk.” [In hindsight] I think that even though [the university] forced me to go through the [match] process, they were already arranging my [residency] position here. (31)

Participants varied in their recollection of the financial cost of their pursuit of US residency, depending on their use of English classes, test preparation courses, and the number of trips for interviews, but most commonly the estimated total was $10 000 (approximately 1 100 000円). Several participants explained moonlighting or borrowing money for this purpose.

## 4 DISCUSSION

Given the challenge of achieving English fluency for many Japanese and its necessity for US clinical residency, it may be anticipated that Japanese with childhood immersion in English would be overrepresented among Japanese IMGs. However, our findings suggest this is not the case. Rather, they indicate that determination and discipline, as demonstrated by the described time management and financial sacrifice, are the more important personal qualities.

It is also clear from the extensive challenges described by our participants that careful planning is necessary to obtain a US residency position. Our data show that uniform decision making or achievements during medical school and clinical residency in Japan are not required. Participants attended a wide range of medical schools that include varied opportunities for electives abroad. They took the American medical licensing examinations and applied for US residency at diverse ages and stages in their medical careers. Surrounding oneself with others striving for similar goals was one strategy to enhance motivation.

Our participants' pathways toward US residency converged most consistently in their establishment of personal connections to the US training system and sometimes to individual US training programs. Participants reconciled this measure in response to the relative anonymity of their academic profiles to US residency committees reviewing many domestic applications, coupled with a Japanese training system lacking a tradition of formal letters of recommendation. The most common, and likely most time‐efficient, method was to use the four recognized institutional liaisons to US training programs. These institutions varied in structure and purpose, but uniformly provided opportunities to visit US training hospitals or to receive letters of recommendation from US trained physicians. For our participants, such opportunities often led directly to interviews for US residency and sometimes offers for residency positions. Significantly, the participants who pursued US training in non–primary care‐related fields that are commonly more competitive,^14^ described moving to the United States, participating in academic activities, and developing personal connections there before applying for US clinical training.

Based on the timing of our study, participants were relatively unaffected by competition from the currently increasing number of US medical school graduates.^21^ The recent rise in residency program applications is accentuating the importance of personal connections and trusted letters of recommendation. Residency directors are unable to review applications as holistically as previously and are changing their methods of resident selection.^13^ Naturally, relevant factors include personal connections and, in the case of Japanese applicants, familiarity with prior Japanese residents and/or a local Japanese patient population. Notably, though USMLE scores are used to filter applications, research repeatedly shows they are not correlated with performance during residency.^22^ This is not surprising since the examinations address medical knowledge but not the remaining core competencies (patient care, practice‐based learning, interpersonal and communication skills, professionalism, and systems‐based practice) evaluated during residency.^23^ Assessing these other qualities of a residency applicant requires spending time with the applicant in a clinical setting. Consistently, residency directors value letters of recommendation, particularly those demonstrating depth of familiarity with the applicant and comparison to other applicants, and evidence supports using structured letters in resident selection.^22^

Unfortunately, obtaining a high‐quality letter of recommendation from an American faculty physician is becoming more difficult. During and since the period of our participants' entrance into US residency, evolving American laws related to patient confidentiality^24^ have prevented the participation in patient care during externships at US hospitals. These developments have decreased the opportunity to demonstrate one's clinical abilities during an externship and, similarly, the opportunity for externships at US teaching hospitals altogether. In turn, it is anticipated that the institutional connections described are becoming more essential. In a current Japanese landscape where interest in US training appears to be sustaining or perhaps increasing,^4^, ^25^ competition to participate in these liaison programs is anticipated to increase.

This study has important limitations. First, as a qualitative study, findings are hypothesis generating and not necessarily generalizable. Second, we included participants who had completed US training up to 11 years prior, but themes did not differ between participants who pursued US clinical training recently vs more remotely. Third, our purposive sampling method utilized referrals from other participants and though we invited participants with potentially contrasting experiences, we may have missed a subset of Japanese IMGs. In turn, our sampling was at risk of bias.

## 5 CONCLUSIONS

Japanese IMGs derive from diverse training programs at diverse ages and commonly without the advantage of prior English fluency. Rather, planning, determination, and discipline appear to be the key personal qualities. In their pursuit of US clinical training, Japanese IMGs emphasize the need for connections to the US medical training system and their pathways commonly converged at a limited number of institutional affiliations.

## CONFLICT OF INTEREST

The authors have stated explicitly that there are no conflicts of interest in connection with this article.

## References

[1] Harden RM . International medical education and future directions: a global perspective. Acad Med. 2006;81(12 Suppl):S22–9. 17086041 10.1097/01.ACM.0000243411.19573.58

[2] Murai M , Kitamura K , Fetters MD . Lessons learned in developing family medicine residency training programs in Japan. BMC Med Educ. 2005;5:33. 16162298 10.1186/1472-6920-5-33 PMC1253513

[3] Saiki T , Imafuku R , Suzuki Y , Ban N . The truth lies somewhere in the middle: swinging between globalization and regionalization of medical education in Japan. Med Teach. 2017;39:1016–22. 28758830 10.1080/0142159X.2017.1359407

[4] Heist BS , Torok HM . Medical migration: a qualitative exploration of the atypical path of Japanese international medical graduates. Med Teach. 2018;40(1):31–9. 28841816 10.1080/0142159X.2017.1370081

[5] Jolly P , Boulet J , Garrison G , Signer MM . Participation in U.S. graduate medical education by graduates of international medical schools. Acad Med. 2011;86(5):559–64. 21436667 10.1097/ACM.0b013e318212de4d

[6] Tsuda T . What we can learn from medication education in the United States: construction of an attractive learning environment. Shinshu Ishi. 2009;57(1):7–18 (In Japanese).

[7] ECFMG to require Medical School Accreditation for international medical school graduates seeking certification beginning in 2023 [press release]. Philadelphia, PA; 2010.

[8] Nara N . Background and outlook for the inauguration of the JACME. JACME (Japan Accreditation Council for Medical Education) Newslett. 2017;1–3 (In Japanese).

[9] JACME (Japan Accreditation Council for Medical Education) Home Page [cited 2020 Feb 18]. Available from https://www.jacme.or.jp (In Japanese).

[10] Walter L . U.S. medical school enrollment rises 30%. Washington, DC: AAMC; 2019.

[11] Ahmed A‐K , Schnatz P , Adashi E . Allopathic and osteopathic medicine unify GME accreditation: a historic convergence. Fam Med. 2017;49:374–7. 28535318

[12] Jolly P , Lischka T , Sondheimer H . Numbers of MD and DO graduates in Graduate Medical Education Programs accredited by the Accreditation Council for Graduate Medical Education and the American Osteopathic Association. Acad Med. 2015;90(7):970–4. 25629946 10.1097/ACM.0000000000000649

[13] Sweet ML , Williams CM , Stewart E , Chudgar SM , Angus SV , Kisielewski M , et al. Internal Medicine Residency Program responses to the increase of residency applications: differences by program type and characteristics. J Grad Med Educ. 2019;11(6):698–703. 31871572 10.4300/JGME-D-19-00194.1 PMC6919188

[14] National Residency Matching Program: results and data 2018 main residency match. 2018 [cited 2020 Feb 20]. Available from https://mk0nrmpcikgb8jxyd19h.kinstacdn.com/wp‐content/uploads/2018/04/Main‐Match‐Result‐and‐Data‐2018.pdf

[15] Ramphul K . International medical graduates in the National Residency Match Game. J Grad Med Educ. 2019;11(4):485. 31440349 10.4300/JGME-D-19-00353.1 PMC6699546

[16] National Residency Matching Program: results and data 2002. 2002 [cited 2020 Feb 20]. Available from https://mk0nrmp3oyqui6wqfm.kinstacdn.com/wp‐content/uploads/2013/08/resultsanddata2002.pdf

[17] Sato T , Nakagawa N , Fujitani S , editors. The road to American clinical study abroad – you can do it!, 4th ed. Nanzandou Publications, Inc.; 2014 (In Japanese).

[18] Heist BS , Torok HM . Japanese International Medical Graduates and the United States clinical training experience: Challenges abroad and methods to overcome them. J Gen Fam Med. 2020 10.1002/jgf2.315 PMC7388660 32742899

[19] Guest G , MacQueen KM , Namey EE . Applied thematic analysis. Thousand Oaks, CA: Sage Publications, Inc.; 2011.

[20] Charmaz K . Grounded theory: objectivist and constructivist methods In: Denzin NK, Lincoln YS, editors. Handbook of qualitative research, 2nd ed. Thousand Oaks, CA: Sage Publications, Inc.; 2000.

[21] AAMC . Matriculants to U.S. medical schools by sex, academic years 1980‐1981 through 2018‐2019. Association of American Medical Colleges; 2018 [cited 2019 Nov 20]. Available from https://www.aamc.org/download/493012/data/factsdatachart3.pdf

[22] Hartman ND , Lefebvre CW , Manthey DE . A narrative review of the evidence supporting factors used by residency program directors to select applicants for interviews. J Grad Med Educ. 2019;11(3):268–73. 31210855 10.4300/JGME-D-18-00979.3 PMC6570461

[23] Accreditation Council for Graduate Medical Education and American Board of Medical Specialties . ACGME competencies: suggested best methods for evaluation [cited 2016 Nov 14]. Available from https://www.partners.org/Assets/Documents/Graduate‐Medical‐Education/ToolTable.pdf

[24] Berwick DM , Gaines ME . How HIPAA harms care, and how to stop it. JAMA. 2018;320(3):229–30. 29926093 10.1001/jama.2018.8829

[25] ECFMG . Standard ECFMG certificates issued in 2017: distribution of recipients by country of medical school and by country of citizenship [cited 2019 Nov 20]. Available from https://www.ecfmg.org/resources/2017CertsbyMedSchoolCountryCitizenshipCountry.pdf

